# Imaging of Reperfused Intramyocardial Hemorrhage with Cardiovascular Magnetic Resonance Susceptibility Weighted Imaging (SWI)

**DOI:** 10.1371/journal.pone.0123560

**Published:** 2015-04-13

**Authors:** James W. Goldfarb, Usama Hasan

**Affiliations:** 1 Department of Research and Education, Saint Francis Hospital, Roslyn, NY, United States of America; 2 Program in Biomedical Engineering, SUNY Stony Brook, Stony Brook, NY, United States of America; 3 New York College of Osteopathic Medicine, Old Westbury, NY, United States of America; Wayne State University, UNITED STATES

## Abstract

**Purpose:**

To report initial experience with TE-averaged susceptibility weighted imaging (SWI) in normal subjects and acute myocardial infarction (AMI) patients for the detection of intramyocardial hemorrhage (IMH).

**Materials and Methods:**

15 healthy control and 11 AMI subjects were studied at 1.5T before contrast agent administration with a dark blood double inversion recovery multiple spoiled gradient-echo sequence. Magnitude, susceptibility weighted and TE-averaged images were reconstructed from raw data. Contrast and signal-difference-to-noise were measured and compared between methods for IMH detection.

**Results:**

There were six patients with microvascular obstruction (MVO) and four patients with IMH detected by TE-averaged SWI imaging. All patients with IMH on SWI scans had MVO on late gadolinium-enhanced (LGE) imaging. There was a three-fold increase in IMH contrast with SWI compared to magnitude images. IMH contrast decreased and signal-to-noise increased with increased TE averages.

**Conclusions:**

TE-averaged SWI imaging is a promising method for myocardial tissue characterization in the setting of AMI for the detection of IMH. Along with gray-scale colormap inversion, it combines not only magnitude and phase information, but also images across TEs to provide a single image sensitive to IMH with characteristics similar to LGE imaging.

## Introduction

Magnetic resonance (MR) offers a noninvasive imaging modality with ongoing technical developments for the assessment of myocardial function, perfusion and tissue characterization [1–4]. In the setting of acute myocardial infarction (AMI), therapeutic [5–8] and spontaneous [9] reperfusion of ischemic myocardium can lead to interstitial intramyocardial hemorrhage (IMH) [10–12] which is associated with microvascular obstruction (MVO) [13,14] and subsequent adverse clinical outcomes [5]. Gadolinium-enhanced imaging [15] is routinely used for the clinical assessment of myocardial perfusion [16] and viability [17]. Imaging without contrast agents (so-called native imaging) can be used in AMI patient for additional myocardial tissue characterization. Native T1 and T2 weighted imaging and quantitative measurements have been reported to detect myocardial edema and depict the myocardial area at risk [18–20]. IMH affects T1, T2 and T2* relaxation as well as susceptibility [21–26] and the feasibility of several MR image contrasts (T1 [27–29], T2 [25,27–40], T2* [25,26,30,38,41,42] and gradient-echo phase [43] has been demonstrated for the depiction of IMH.

Susceptibility weighted imaging (SWI) [44,45] uses a type of image contrast different from traditional spin density, T1 or T2 weighted MR imaging. SWI typically uses a flow compensated, long gradient recalled echo pulse sequence to acquire complex images and then emphasized susceptibility differences between tissues contained in image phase. Magnitude and phase data are combined to produce an enhanced contrast magnitude image which is sensitive to venous blood, hemorrhage and iron storage. The aim of SWI is to enhance the image contrast between tissues with different susceptibilities for better visualization. Original SWI was a single gradient-echo sequence, but has been extended to multiple echoes for improvements in contrast and signal-to-noise (SNR) using echo combination [46–49].

In a recent publication [43], we showed that IMH significantly reduces image phase below normal variations. In the present work, we report our initial experience with myocardial SWI imaging (combined gradient-echo magnitude and phase imaging) in normal subjects and AMI patients for the detection of IMH. We propose TE image averaging and gray-scale inversion as a means of providing a single image with good image SNR and excellent contrast for the detection of IMH. Additionally, we compared single-echo SWI and TE-averaged SWI quantitatively for image SNR and contrast characteristics.

## Materials and Methods

Study data from a previous published report [43] which studied gradient-echo image phase was used to generate SWI images. LV functional measures and infarct sizes are listed in this publication. Twenty-six individuals underwent MR imaging at 1.5T using a standard body matrix phased array coil (Magnetom Siemens Avanto, Erlangen, Germany). The coil consisted of four clusters of three anterior and posterior elements. Elements were combined with the Auto (CP) coil mode and sampled as four clusters. Eleven subjects were enrolled at 3 days after their first myocardial infarction (age = 55.0 ± 11.5 years, range = 38.1–79.0) and fifteen were healthy control subjects (age = 61.7 ± 8.9 years, range = 41.9–76.4). AMI patients volunteered for this research study prior to hospital discharge after successful emergent reperfusion therapy with stent placement. Each subject underwent imaging according to a protocol approved by the institutional review board. All subjects signed an institutional review board approved, Health Insurance Portability and Accountability Act compliant consent form prior to study initiation.

### MR Imaging

Acquisitions were performed during suspended respiration at end-expiration. Oxygen (2 l/min via a nasal cannula) was provided to improve breath-holding. For each part of the study, identical slice locations were used consisting of 7–10 parallel short-axis slices spanning the LV myocardium from the base to apex and three LV long axis views (two-, three, and four chamber). Before contrast agent administration, a dark blood double inversion recovery multiple spoiled gradient-echo sequence (1 slice per breathhold, repetition time = 20 ms; 12 echo times, 2.4–15.5 ms (1.2 ms spacing), flip angle = 20 degrees, bandwidth = 1860 Hz/pixel, in-plane spatial resolution = 2.3 x 1.7 mm^2^, slice thickness = 8 mm, velocity compensation in read and slice) was used for SWI imaging. Imaging was performed in mid-diastole using a prospectively gated segmented acquisition of k-space over 13 heartbeats with ECG gating using a signal from leads placed on the subject's chest. Raw k-space data were saved to the scanners hard disk and transferred to a personal computer for offline image reconstruction. A phase sensitive inversion recovery gradient-echo pulse sequence (1 slice per breathhold, repetition time = 8.7 ms; echo time = 3.4 ms; flip angle = 25 degrees, bandwidth = 130 Hz/pixel, in-plane spatial resolution, 1.7 x 1.3 mm^2^, slice thickness = 8 mm) was used for late gadolinium enhanced (LGE) infarct imaging [50]. LGE imaging was commenced 15 minutes after bolus contrast agent administration (0.15 mmol/kg gadopentetate dimeglumine, Magnevist, Bayer Healthcare, Wayne, NJ). The inversion time parameter was manually adjusted to minimize the signal of viable myocardium (250–350 ms) at the start of LGE acquisitions.

### Image Reconstruction

Offline SWI image reconstruction was performed using Matlab R2009b (Mathworks, Natick, MA) from saved raw data files for both long and short axis images. Three classes of image reconstruction were performed: 1) standard magnitude and high-pass filtered phase 2) SWI and 3) echo time (TE) averaged.

1. **Standard Magnitude and High-pass Filtered Phase Image Reconstruction.** Images were independently reconstructed for each TE and coil element. Complex images were reconstructed using Matlab’s two-dimensional discrete Fourier transform function (fft2) and full resolution magnitude and phase images were extracted. Next, a complex low pass filtered (LPF) image using an N = 64 point radial squared hanning filter $LPF\;\left(kx,ky\right){\;=\;[0.5\;(1-cos\left(2\pi \frac{\sqrt{{kx}^{2}{+ky}^{2}}}{N-1}\right)]}^{2}$ was reconstructed by filtering k-space data and Fourier transform. High-pass filtered phase images were then calculated by dividing the low pass filtered image into the full resolution complex image and then extracting the phase component [44]. Individual coil element magnitude images were combined using a sum-of-squares reconstruction [51].
2. **Susceptibility Weighted Image (SWI) Reconstruction.** SWI images for each TE were constructed with 3 and 6 phase mask multiplications (N).
  $$SWI\left(x,y\right)=\left|I\left(x,y\right)\right|*Phase\;mask{\left(x,y\right)}^{N}$$
  The phase mask was computed to emphasize negative phase:
  $$Phase\;mask\left(x,y\right)=\{\begin{array}{cc} \begin{array}{c} \left(\pi +\varphi \left(x,y\right)\right)/\pi \end{array} & -\pi <\varphi \left(x,y\right)<0\\ 1 & otherwise \end{array}$$
3. **TE-Averaged Image Reconstruction.** To improve signal-to-noise, magnitude and SWI images were averaged across echo times (nTE = number of TE averages). 11 averaged images were constructed by averaging the longest echo time images. The longer echo times have the lowest SNR and greatest IMH contrast. Therefore averaged images consisted of (nTE = 2) = TE11+TE12, (nTE = 3) = TE10+ TE11+ TE12, (nTE = 4) = TE9+ TE10+ TE11+ TE12, etc. nTE = 1 represents the longest TE image without averaging.

T2* measurements were performed from magnitude images using ImageJ for region-of-interest (ROI) measurements and Matlab for curve fitting. ROIs were drawn on the short-axis images (basal, mid-ventricular and apical) according to the 16-segment AHA model [52]. For each ROI, the mean image intensity for each TE was computed and a T2* decay curve was constructed. Each measured decay curve was fitted with an exponential function: S = So exp(-TE/T2*) + C; where S is the image signal intensity, So and C are constants, TE is the echo time and T2* is the transverse relaxation time.

### Image Analysis

Average signal intensity was measured from identical region-of-interests for each reconstruction in the hemorrhagic infarct (identified as a hypointense lesion) and adjacent myocardium. The noise standard deviation was estimated from an ROI without signal (either outside of the body or in the lung cavity). Contrast and signal-difference-to-noise (SDNR) [53] were calculated from these measurements for source magnitude images, SWI images and TE-averaged images. Contrast = (S1-S2)/S1 and SNDR = (S1-S2)/STD, where S1 and S2 are mean image intensities and STD is and estimate of the noise standard deviation. Analysis of variance (ANOVA) and Student’s t-test was used to determine significant differences between reconstruction methods and number of TE averages as appropriate. A p-value less than 0.05 was used to determine significant differences.

A single reader blindly reviewed TE-averaged SWI images for artifacts and recorded the number of control subjects and AMI patient with artifacts and their anatomical location. Using a semi-automatic detection algorithm, a signal intensity threshold of fifty percent of the maximum myocardial signal was applied to quantify the total LGE infarct volume from short-axis images [54]. Discrete areas of hypointensity within LGE were considered to be MVO. Although under the fifty percent signal intensity threshold, these pixels were included in the calculation of LGE infarct volume. Volumes of IMH and MVO were measured using visual intensity thresholding. LGE, IMH and MVO volumes are reported as a percentage of LV myocardial volume. For the analyses of T2* and segments with IMH detected with TE-averaged SWI, repeated measures ANOVA and mixed model analysis was used. Marginal means and standard deviations reported.

## Results

There were nine transmural and three subendocardial MIs. Culprit lesions were located in the left anterior descending coronary artery [n = 3], left circumflex coronary artery [n = 3] and right coronary artery [n = 5]. Peak cardiac serum biomarkers were Creatine Phosphokinase (CPK) (n = 10) = 1612.9 ± 813.5 (u/l), Creatine Kinase-MB (CK-MB) (n = 10) = 123.0 ± 95.7 (ng/ml), MB Relative Index (n = 10) = 7.3 ± 3.4 (%) and Troponin I (n = 8) = 25.4± 29.5 (ng/ml).

**Fig 1:**
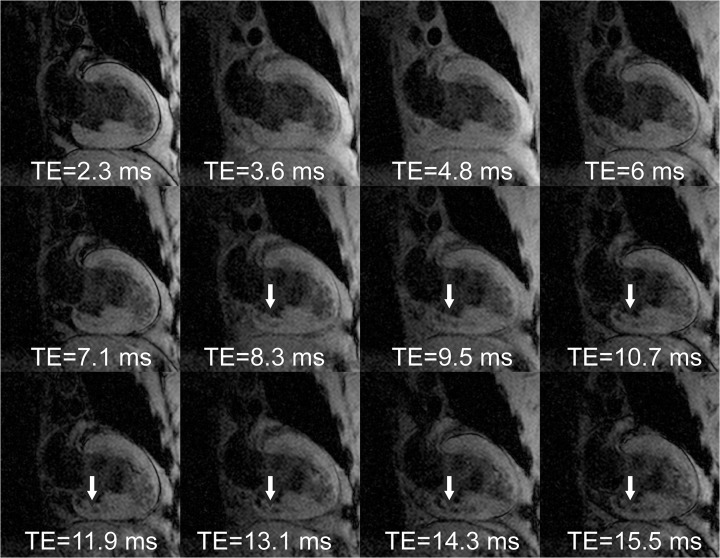
Source magnitude multiple gradient-echo images from a patient with an inferior myocardial infarction at 3 days after emergent revascularization and stenting of a right coronary artery occlusion. A hypointense lesion is seen in the basal inferior wall. T2* weighting increases with echo time improving intramyocardial hemorrhage (IMH) lesion (arrows) contrast, but signal-to-noise loss at longer echo times markedly reduces image quality.

**Fig 2:**
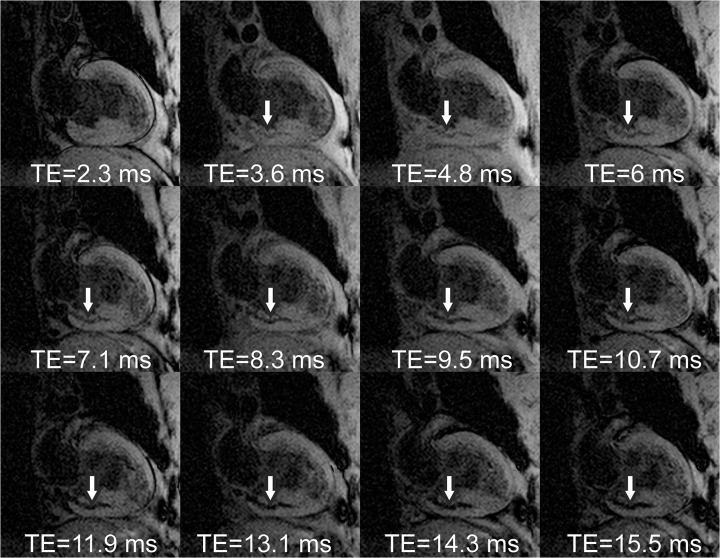
Susceptibility weighted (SWI) images from a patient with an inferior myocardial infarction at 3 days after emergent revascularization and stenting of a right coronary artery occlusion. Images combine magnitude and phase for each individual echo time. Note the increased IMH lesion (arrows) contrast and definition compared to source magnitude images (Fig 1).

**Fig 3:**
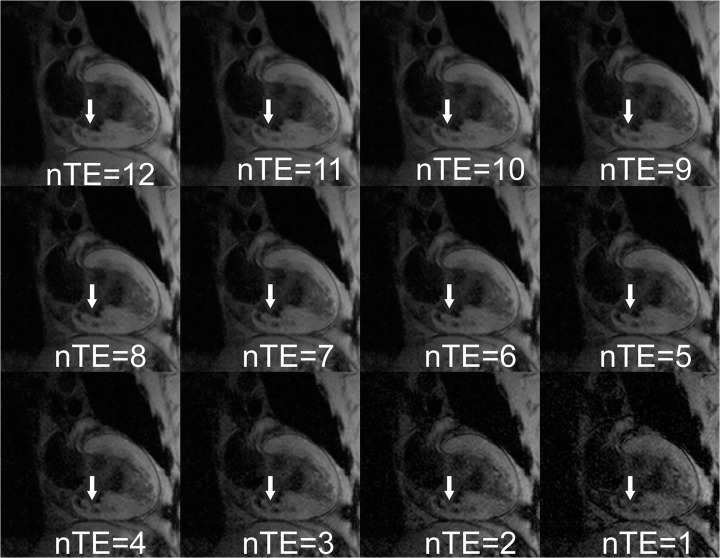
TE-averaged magnitude images from a patient with an inferior myocardial infarction at 3 days after emergent revascularization and stenting of a right coronary artery occlusion. Images are averaged over the nTE longest source echo time images (Fig 1). Note the improved SNR, but decreased IMH lesion (arrows) definition compared to source images.

**Fig 4:**
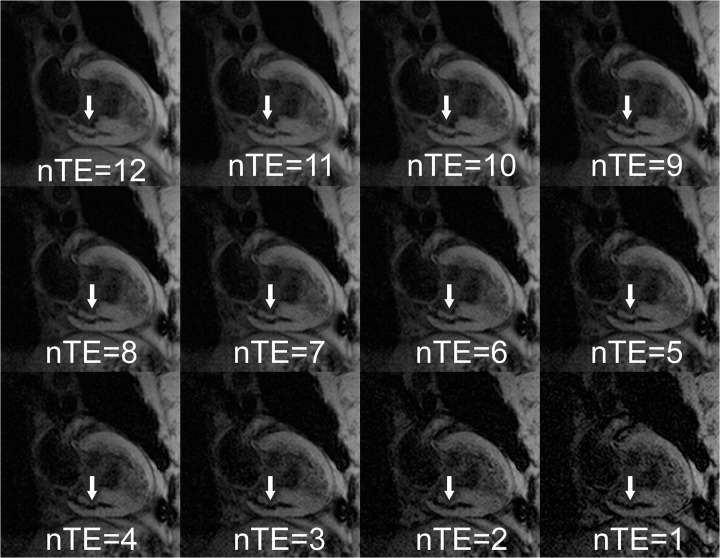
TE-averaged susceptibility weighted (SWI) images from a patient with an inferior myocardial infarction at 3 days after emergent revascularization and stenting of a right coronary artery occlusion. Images are averaged over the nTE longest SWI images (Fig 2). Note the improved SNR and loss of contrast increased number of echo time averages (NTE). nTE = 9 was quantitatively shown to have the best SNR across patients with good hemorrhage contrast.

**Fig 5:**
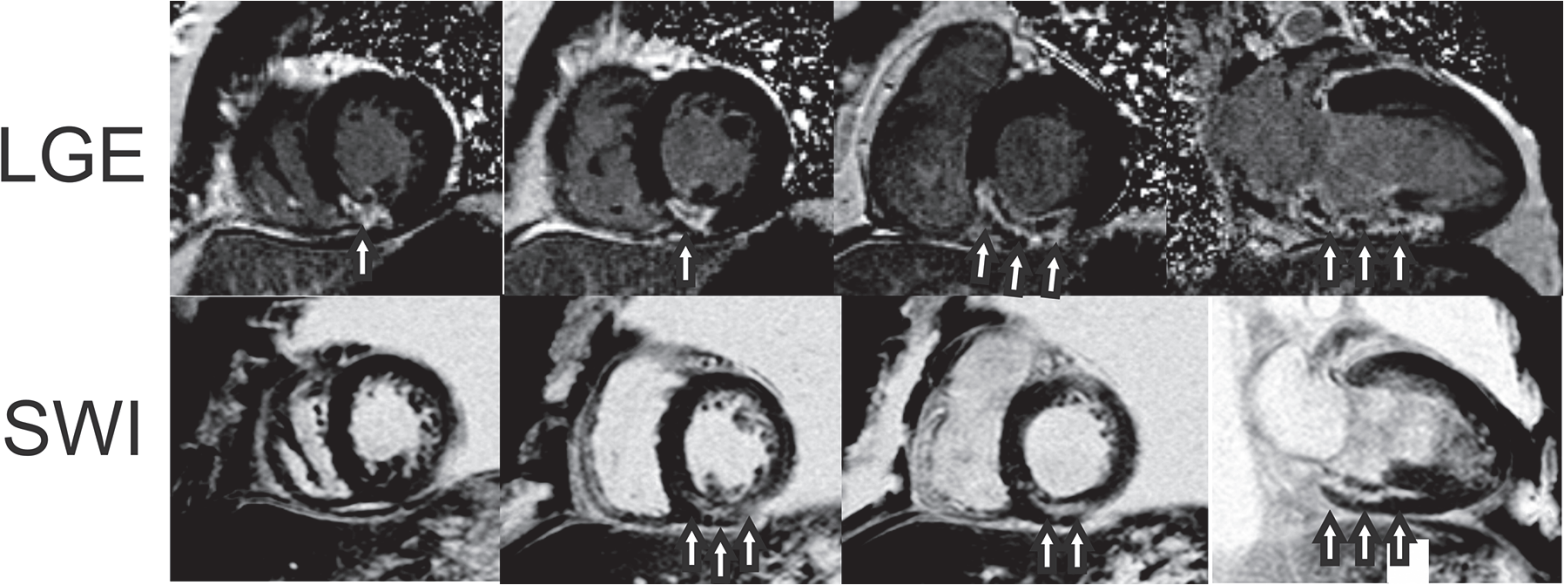
Short axis and VLA late gadolinium-enhanced (LGE) and echo-combined (nTE = 9) susceptibility weighted (SWI) images from a patient with an inferior myocardial infarction at 3 days after emergent revascularization and stenting of a right coronary artery occlusion. SWI images are displayed with an inverted grayscale colormap to show abnormal tissue as hyperintense, similar to LGE images. LGE images show circumscribed hyperenhancement of the mid to basal inferior wall infarct (arrows) with microvascular obstruction (MVO) (top row, arrows). SWI images obtained before contrast agent administration show a mid-wall hyperintense lesion consistent with intramyocardial hemorrhage (bottom row, arrows).

Results from a representative AMI subject are given in Figs 1–5. Source magnitude images (Fig 1) show an IMH lesion in the mid to basal inferior wall. SWI images (Fig 2) show better lesion definition via improved image contrast across all echo times. The IMH lesion is best defined at TE = 15.5ms, but image quality is severely degraded by increased noise giving it a cloudy appearance. TE-averaging (Figs 3 and 4) improves SNR for both magnitude and SWI images, but IMH contrast is better in SWI images, (see Fig 4, nTE = 9). Grayscale colormap inversion of echo combined SWI images (Fig 5) yields images characteristics similar to LGE imaging; normal myocardium is dark, with bright fat and blood. In this patient with MVO, IMH is seen as a hyperintense lesion in the inferior wall in the same segment as MVO.

**Fig 6:**
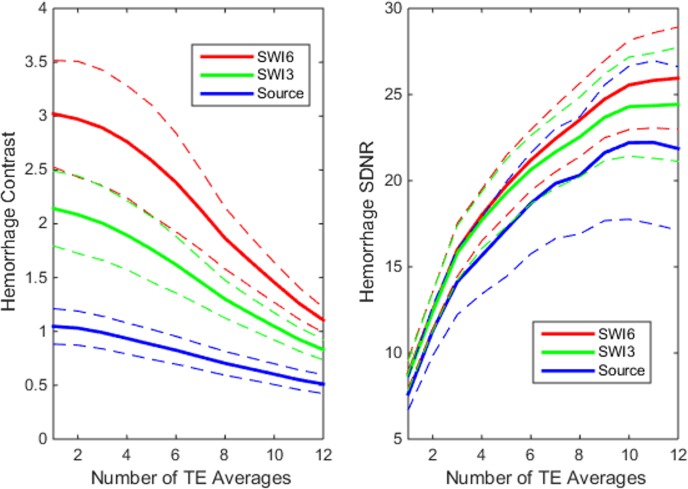
Analysis of Hemorrhage contrast and signal-to-noise. Graphs show the effect of increased TE-averaging on hemorrhage contrast and signal difference to noise ratio (SDNR) for susceptibility weighted (SWI) and source magnitude images. Contrast decreases with more TE averages (nTE), while SDNR increases up to nTE = 9. Six phase mask multiplies (SWI6) when compared to three (SWI3) provided better contrast and SDNR.

Quantitative results from ROI measurements displayed in Fig 6 show the reduction in IMH contrast and increase in IMH SDNR with increased averages (p<0.001). There was a three-fold increase in IMH contrast with SWI compared to source images. Contrast increased when comparing six vs. three phase mask multiplications (p<0.01). Contrast decreased and signal-to-noise increased with increased TE averages. SDNR increased with TE averages up to nTE = 9 (p = 0.07). When comparing unaveraged single echo magnitude source and SWI images, SDNR increased 75.1% and 77.5% respectively as echo time increased from TE = 2.3 to 15.5ms in this study. Both were always lower than TE-averaged image SDNR irrespective of the number of averages.

**Fig 7:**
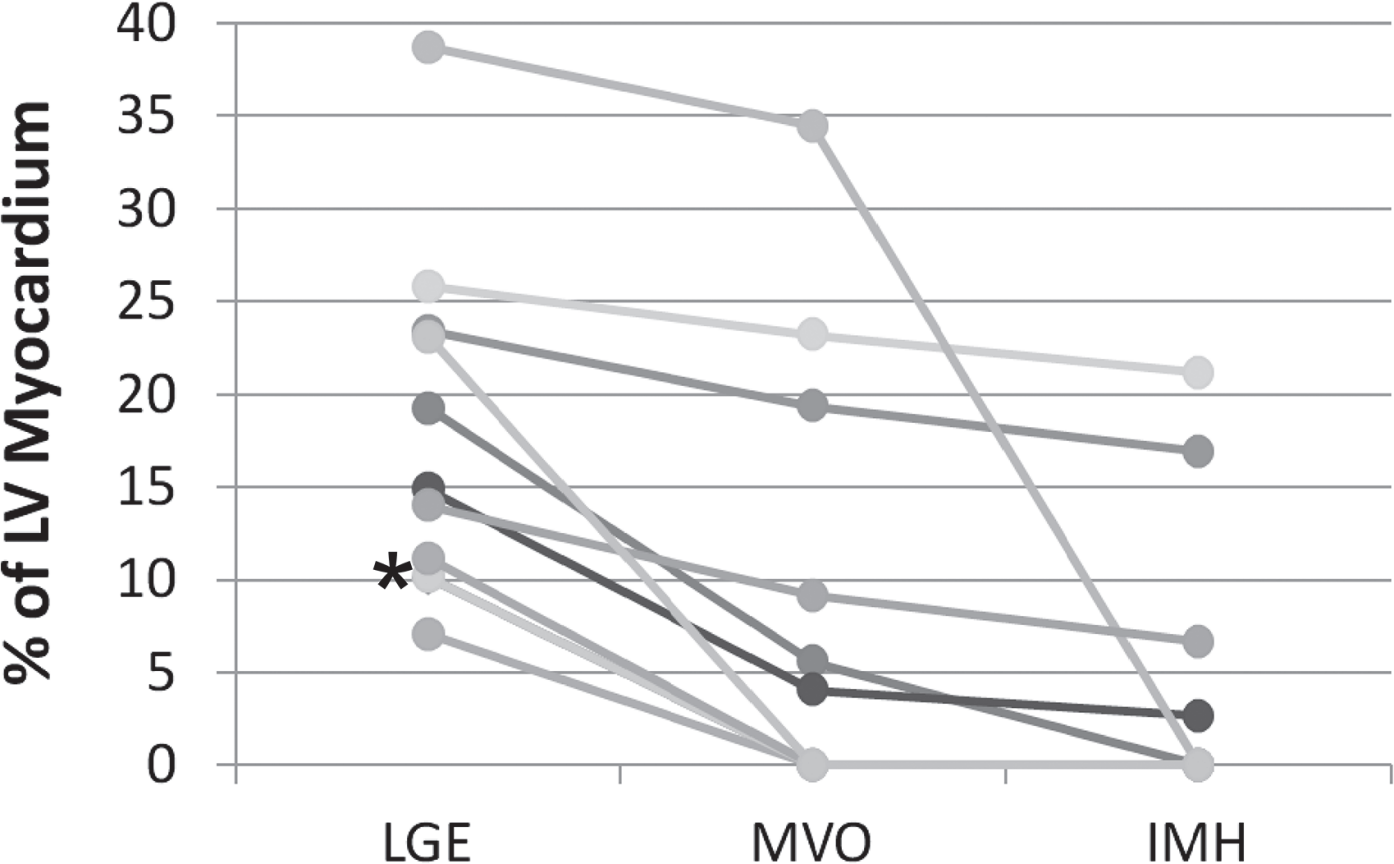
Comparison among LGE, MVO and IMH volumes. Graph shows the left ventricular percentage of late gadolinium enhancement (LGE), microvascular obstruction (MVO) and intramyocardial hemorrhage (IMH) for acute myocardial infarction study participants. * denotes two subjects with similar volume which overlap in the graph.

**Fig 8:**
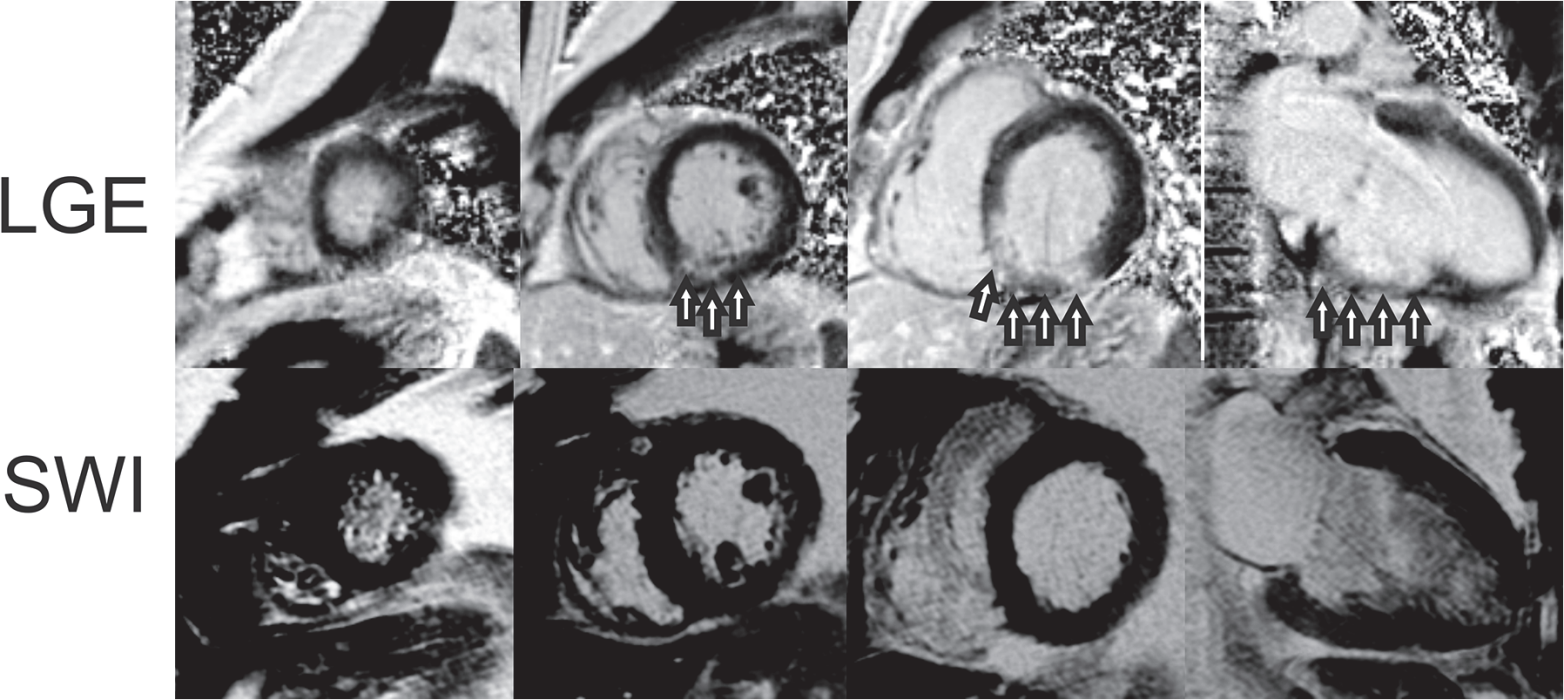
Example study images from an acute myocardial infarction patient without intramyocardial hemorrhage (IMH). Images from a patient with an inferior wall myocardial infarction. LGE images show transmural hyperenhancement (top row arrows) of the inferior wall. Precontrast SWI images show no hyperintense lesions indicative of IMH (bottom row).

**Fig 9:**
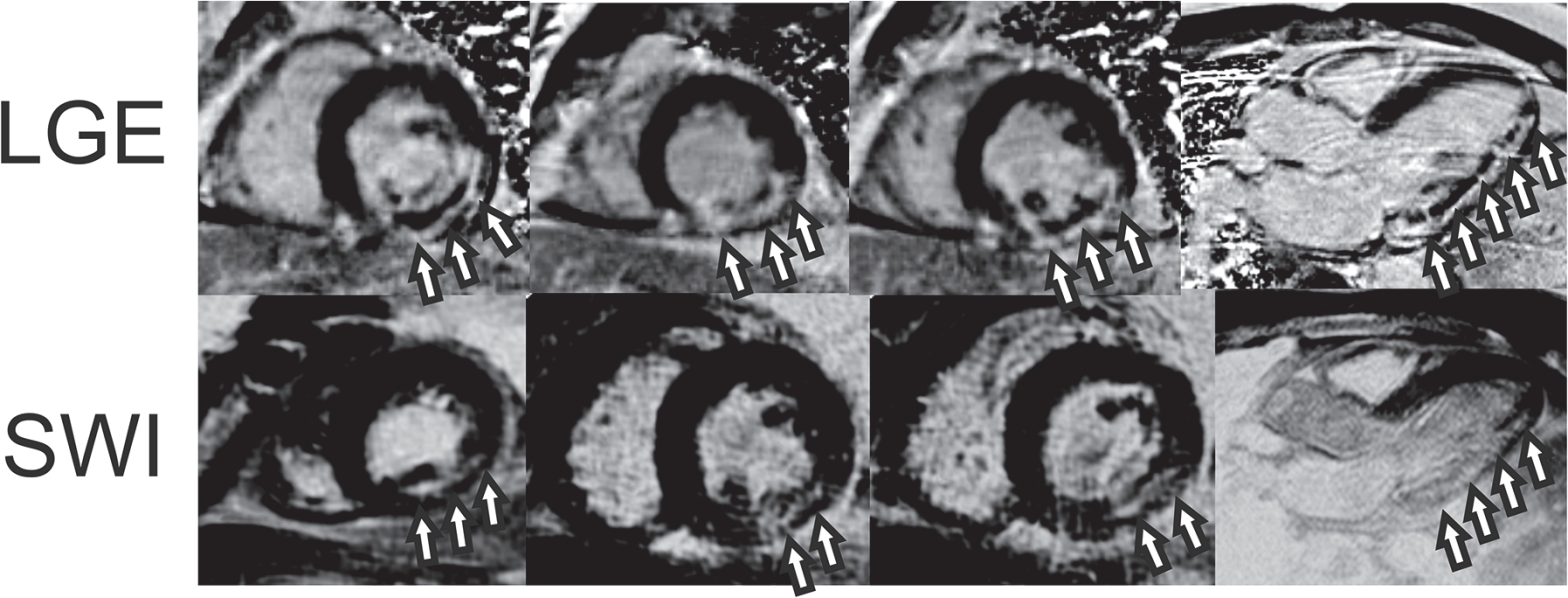
Example study images from an acute myocardial infarction patient with intramyocardial hemorrhage (IMH). Short-axis and three-chamber images from a patient with an inferior lateral wall myocardial infarction. LGE images show transmural infarction with MVO (Top row, arrows). Precontrast SWI images show a hyperintense lesion consistent with IMH in the same area as MVO defined by LGE imaging (bottom row, arrows).

**Fig 10:**
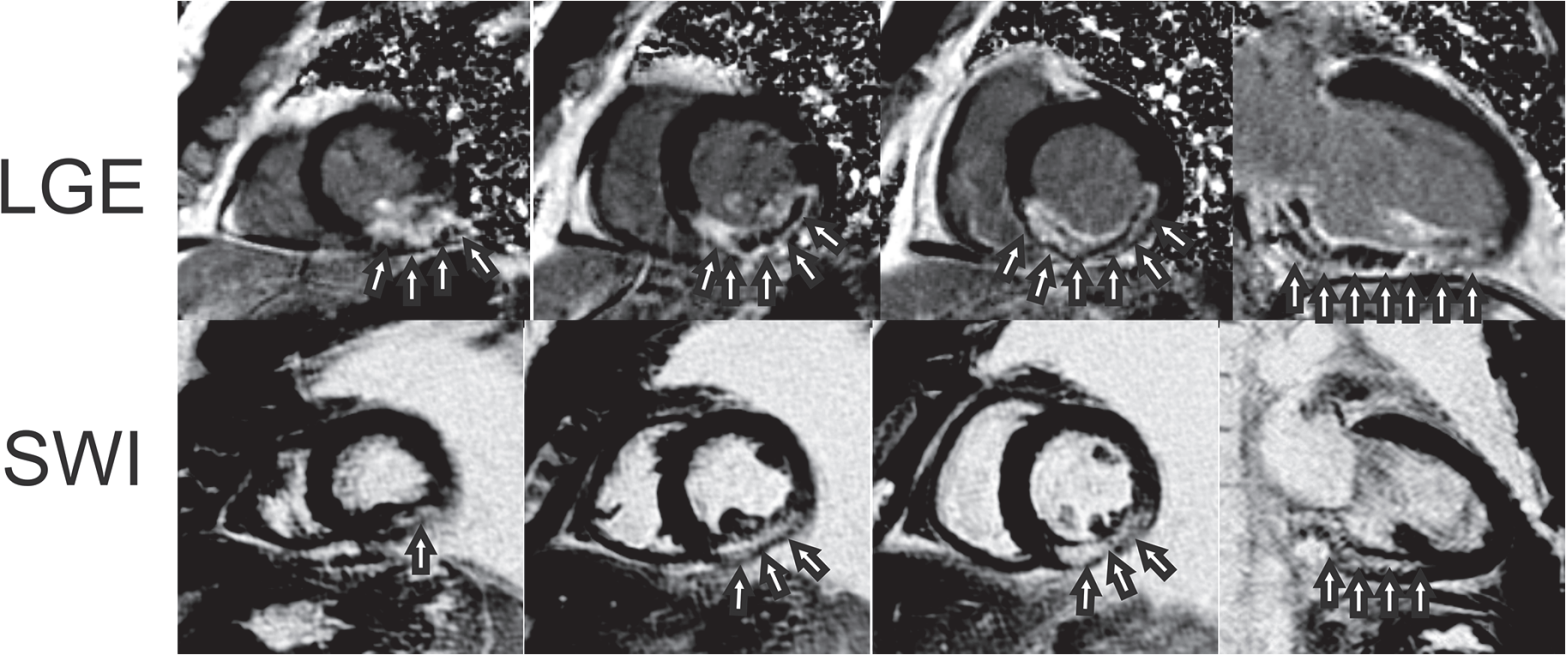
Example study images from an acute myocardial infarction patient with intramyocardial hemorrhage (IMH). Short-axis and two chamber images from a patient with an acute inferior lateral wall myocardial infarction. LGE images show circumscribed transmural infarction with MVO (Top row, arrows). Precontrast SWI images show a hyperintense lesion consistent with IMH in the same area as MVO defined by LGE imaging (bottom row, arrows).

There were six patients with microvascular obstruction and four patients with intramyocardial hemorrhage detected by TE-averaged SWI imaging. All patients with IMH on SWI scans had MVO on LGE scans. Results of IMH and MVO volume measurements are displayed in Fig 7. The trend was that larger infarcts had MVO and IMH, but this was not true in all cases as the largest infarct had no evidence of IMH. Three additional patients are shown in Figs 8, 9 and 10. The first without IMH (Fig 8 and two patients with IMH (Figs 9 and 10). Excellent IMH definition is realized with similar positive image contrast when compared to LGE images.

**Fig 11:**
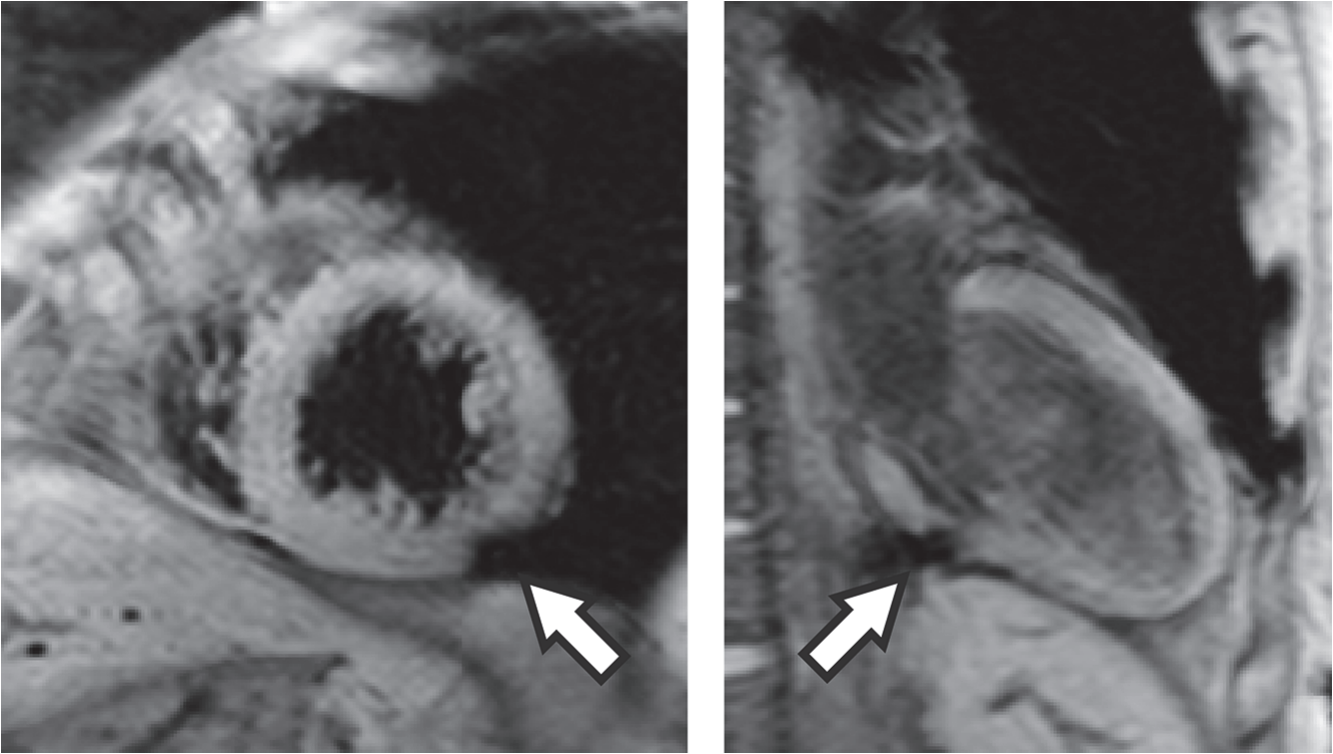
Artifacts observed in TE-averaged susceptibility weighted imaging (SWI). Image artifacts from coronary artery veins are shown (arrows) from two patients in short axis and two-chamber views.

Fig 11 shows a common artifact found in TE-averaged SWI images resulting from the coronary vein. 6/15 (40%) control subjects and 3/11 (27%) of AMI patients had at least one image with this artifact. Although common among patients, the artifact rarely affected more than one slice orientation. Six two chamber, three short axis and one three chamber orientations were affected, allowing interpretation of the myocardial segments in other orientations without image artifacts.

There was a no significant difference in segmental T2* between LGE absent (34.1 ± 0.7 ms) and LGE positive segments without IMH (T2* = 35.3 ± 1.2 ms). But both were significantly different with segments with IMH (T2* = 16.2 ± 3.3 ms) detected by TE-averaged SWI imaging (p<0.001), showing that TE-averaged SWI imaging is consistent with T2* calculations.

## Discussion

This study has demonstrated the utility of SWI imaging for myocardial tissue characterization in the acute phase of myocardial infarction. SWI depicted myocardium in areas of MVO indicative of IMH. When compared to magnitude imaging, SWI provided incremental improvements in IMH contrast and TE-averaging provided incremental improvements in image SNR. Combined with grayscale colormap inversion, TE-averaged SWI imaging provided a high image contrast and SNR method for the depiction of IMH with characteristics similar to LGE infarct imaging.

There are a number of MR methods for the detection of IMH with encouraging reports in the literature. T1 weighted imaging is sensitive not only to IMH [24] but also to myocardial edema [19] and intramyocardial fat [55]. T2-weighted imaging [18] depends on the hyperintense signal from myocardial edema and depiction of IMH as a hypointense core [28,29,31,35,36,38]. Both HPF phase imaging [43] and T2* imaging [25,26,30,38,41,42] are closely related to SWI imaging. T2* weighted imaging with a single long TE is often compromised by dropout artifacts in the inferior and lateral walls, SNR and motion degradation. T2* mapping combines images from multiple TEs into a quantitative map where a normal T2* threshold could be applied, but also can suffer from image quality artifacts. HPF phase imaging produces a phase image which is also quantitative, but is typically not clinically viewed. TE-averaged SWI should be viewed as an incremental improvement to T2*-weighted imaging of IMH as it utilizes the same pulse sequence, but provides better contrast via magnitude and phase combination and averaging over TEs to improve SNR. SWI similar to T2* and T2 techniques detect IMH via a low contrast signal mechanism. Detecting hypointense IMH could be complicated by other low intense structures such as the LV cavity. IMH is typically seen within the LV myocardium and in this study was always surrounded by high contrast myocardium. SWI image artifacts can results from sources with susceptibility differences. Deoxygenated blood in the coronary sinus was a frequent source of an artifact identified in this study. The coronary vein can be distinguished from IMH due to its predictable location outside of the LV myocardium.

Higher main magnetic field strengths (3T or 7T) have the advantage of better susceptibility weighting, but may have more artifacts. Optimal field strength for IMH detection would have to be determined, but preliminary results obtained in this paper support the use of 1.5T SWI imaging for the detection of IMH. Gray scale inversion of SWI images yields positive contrast with bright injured myocardium and dark or nulled viable myocardium. Image contrast could improve adoption with bright IMH similar to infarcted myocardium in LGE imaging, but could also cause confusion between the two imaging techniques. Another advantage of this technique and motivation of this work is the combination of multiple (in this case 24) magnitude and phase images into a single image. Similar to T2* mapping. TE-averaged SWI imaging yields fewer images through image information combination to improve physician evaluation speed.

The results of this study should be considered in light of some limitations. Limitations of this study include the small study size, predominantly male study population. Patients in this study were physiologically stable and without contraindication to MR imaging. The patient population must be considered when interpreting the data and may not be representative of a larger population. IMH was not seen in normal volunteers or in segments without LGE. This study has demonstrated the feasibility of IMH detection with TE-averaged SWI imaging, but future larger studies are needed to determine its widespread performance and accuracy across a wide range of infarct ages and types. Optimal imaging parameters for routine clinical imaging have not been determined in this study. The optimal longest TE, number of echo times as well as the necessity of velocity compensation in imaging myocardium should be further investigated. We did not investigate lower bandwidth acquisitions which would increase signal-to-noise at the expense of a longer sampling intervals during the cardiac cycle and hence did not determine the optimal bandwidth for cardiac SWI imaging. Semi-automated image analysis was used and manual drawing of endo- and epi-cardial borders as well as intensity threshold may be operator dependent.

In conclusion, TE-averaged SWI imaging is a promising method for myocardial tissue characterization in the setting of AMI for the detection of IMH. Along with gray-scale inversion, it combines not only magnitude and phase information, but also images across several TEs to provide native images sensitive to IMH with characteristics similar to LGE imaging.

## References

[1] El Aidi H, Adams A, Moons KG, Den Ruijter HM, Mali WP, Doevendans PA, et al (2014) Cardiac magnetic resonance imaging findings and the risk of cardiovascular events in patients with recent myocardial infarction or suspected or known coronary artery disease: a systematic review of prognostic studies. J Am Coll Cardiol 63: 1031–1045. 10.1016/j.jacc.2013.11.048 24486280

[2] Rajiah P, Desai MY, Kwon D, Flamm SD (2013) MR imaging of myocardial infarction. Radiographics 33: 1383–1412. 10.1148/rg.335125722 24025931

[3] Salerno M, Kramer CM (2013) Advances in parametric mapping with CMR imaging. JACC Cardiovasc Imaging 6: 806–822. 10.1016/j.jcmg.2013.05.005 23845576 PMC4073213

[4] Perazzolo Marra M, Lima JA, Iliceto S (2011) MRI in acute myocardial infarction. Eur Heart J 32: 284–293. 10.1093/eurheartj/ehq409 21112897

[5] Wu KC, Zerhouni EA, Judd RM, Lugo-Olivieri CH, Barouch LA, Schulman SP, et al (1998) Prognostic significance of microvascular obstruction by magnetic resonance imaging in patients with acute myocardial infarction. Circulation 97: 765–772. 9498540 10.1161/01.cir.97.8.765

[6] Wu KC (2012) CMR of microvascular obstruction and hemorrhage in myocardial infarction. J Cardiovasc Magn Reson 14: 68 10.1186/1532-429X-14-68 23021401 PMC3514126

[7] Saeed M, Hetts S, Wilson M (2010) Reperfusion injury components and manifestations determined by cardiovascular MR and MDCT imaging. World J Radiol 2: 1–14. 10.4329/wjr.v2.i1.1 21160735 PMC2999314

[8] Saeed M, Martin AJ, Saloner D, Do L, Wilson M (2010) Noninvasive MR characterization of structural and functional components of reperfused infarct. Acta Radiol 51: 1093–1102. 10.3109/02841851.2010.520025 21062130

[9] Vargas-Barron J, Gonzalez-Pacheco H, Melendez-Ramirez G, Roldan FJ, Damas-De Los Sasntos F, Meave-Gonzalez A, et al (2014) Intramyocardial hemorrhage in spontaneously reperfused myocardial infarction. Rev Invest Clin 66: 107–112. 24960319

[10] Basso C, Thiene G (2006) The pathophysiology of myocardial reperfusion: a pathologist's perspective. Heart 92: 1559–1562. 16547203 10.1136/hrt.2005.086959 PMC1861239

[11] Garcia-Dorado D, Theroux P, Solares J, Alonso J, Fernandez-Aviles F, Elizaga J, et al (1990) Determinants of hemorrhagic infarcts. Histologic observations from experiments involving coronary occlusion, coronary reperfusion, and reocclusion. Am J Pathol 137: 301–311. 2386198 PMC1877616

[12] Yellon DM, Hausenloy DJ (2007) Myocardial reperfusion injury. N Engl J Med 357: 1121–1135. 17855673 10.1056/NEJMra071667

[13] Schwartz RS (2005) Microvascular obstruction in acute coronary syndromes: onward to a new therapeutic target. Catheter Cardiovasc Interv 66: 170–172. 16142803 10.1002/ccd.20510

[14] Rochitte CE, Lima JA, Bluemke DA, Reeder SB, McVeigh ER, Furuta T, et al (1998) Magnitude and time course of microvascular obstruction and tissue injury after acute myocardial infarction. Circulation 98: 1006–1014. 9737521 10.1161/01.cir.98.10.1006

[15] Edelman RR (2004) Contrast-enhanced MR imaging of the heart: overview of the literature. Radiology 232: 653–668. 15284429 10.1148/radiol.2323031558

[16] Greenwood JP, Maredia N, Radjenovic A, Brown JM, Nixon J, Farrin AJ, et al (2009) Clinical evaluation of magnetic resonance imaging in coronary heart disease: the CE-MARC study. Trials 10: 62 10.1186/1745-6215-10-62 19640271 PMC3224948

[17] Simonetti OP, Kim RJ, Fieno DS, Hillenbrand HB, Wu E, Bundy JM, et al (2001) An improved MR imaging technique for the visualization of myocardial infarction. Radiology 218: 215–223. 11152805 10.1148/radiology.218.1.r01ja50215

[18] Abdel-Aty H, Simonetti O, Friedrich MG (2007) T2-weighted cardiovascular magnetic resonance imaging. J Magn Reson Imaging 26: 452–459. 17729358 10.1002/jmri.21028

[19] Goldfarb JW, Arnold S, Han J (2007) Recent myocardial infarction: assessment with unenhanced T1-weighted MR imaging. Radiology 245: 245–250. 17885192 10.1148/radiol.2451061590

[20] Ferreira VM, Piechnik SK, Dall'Armellina E, Karamitsos TD, Francis JM, Choudhury RP, et al (2012) Non-contrast T1-mapping detects acute myocardial edema with high diagnostic accuracy: a comparison to T2-weighted cardiovascular magnetic resonance. J Cardiovasc Magn Reson 14: 42 10.1186/1532-429X-14-42 22720998 PMC3424120

[21] Lotan CS, Bouchard A, Cranney GB, Bishop SP, Pohost GM (1992) Assessment of postreperfusion myocardial hemorrhage using proton NMR imaging at 1.5 T. Circulation 86: 1018–1025. 1516171 10.1161/01.cir.86.3.1018

[22] Kali A, Tang RL, Kumar A, Min JK, Dharmakumar R (2013) Detection of acute reperfusion myocardial hemorrhage with cardiac MR imaging: T2 versus T2. Radiology 269: 387–395. 10.1148/radiol.13122397 23847253 PMC3807083

[23] Zia MI, Ghugre NR, Connelly KA, Strauss BH, Sparkes JD, Dick AJ, et al (2012) Characterizing myocardial edema and hemorrhage using quantitative T2 and T2* mapping at multiple time intervals post ST-segment elevation myocardial infarction. Circ Cardiovasc Imaging 5: 566–572. 22744938 10.1161/CIRCIMAGING.112.973222

[24] Pedersen SF, Thrysoe SA, Robich MP, Paaske WP, Ringgaard S, Botker HE, et al (2012) Assessment of intramyocardial hemorrhage by T1-weighted cardiovascular magnetic resonance in reperfused acute myocardial infarction. J Cardiovasc Magn Reson 14: 59 22935462 10.1186/1532-429X-14-59 PMC3457904

[25] O'Regan DP, Ahmed R, Karunanithy N, Neuwirth C, Tan Y, Durighel G, et al (2009) Reperfusion hemorrhage following acute myocardial infarction: assessment with T2* mapping and effect on measuring the area at risk. Radiology 250: 916–922. 10.1148/radiol.2503081154 19164125

[26] O'Regan DP, Ariff B, Neuwirth C, Tan Y, Durighel G, Cook SA (2010) Assessment of severe reperfusion injury with T2* cardiac MRI in patients with acute myocardial infarction. Heart 96: 1885–1891. 10.1136/hrt.2010.200634 20965977

[27] Basso C, Corbetti F, Silva C, Abudureheman A, Lacognata C, Cacciavillani L, et al (2007) Morphologic validation of reperfused hemorrhagic myocardial infarction by cardiovascular magnetic resonance. Am J Cardiol 100: 1322–1327. 17920380 10.1016/j.amjcard.2007.05.062

[28] Foltz WD, Yang Y, Graham JJ, Detsky JS, Wright GA, Dick AJ (2006) MRI relaxation fluctuations in acute reperfused hemorrhagic infarction. Magn Reson Med 56: 1311–1319. 17089360 10.1002/mrm.21079

[29] Jackowski C, Christe A, Sonnenschein M, Aghayev E, Thali MJ (2006) Postmortem unenhanced magnetic resonance imaging of myocardial infarction in correlation to histological infarction age characterization. Eur Heart J 27: 2459–2467. 16973689 10.1093/eurheartj/ehl255

[30] Asanuma T, Tanabe K, Ochiai K, Yoshitomi H, Nakamura K, Murakami Y, et al (1997) Relationship between progressive microvascular damage and intramyocardial hemorrhage in patients with reperfused anterior myocardial infarction: myocardial contrast echocardiographic study. Circulation 96: 448–453. 9244211 10.1161/01.cir.96.2.448

[31] Beek AM, Nijveldt R, van Rossum AC (2010) Intramyocardial hemorrhage and microvascular obstruction after primary percutaneous coronary intervention. Int J Cardiovasc Imaging 26: 49–55. 10.1007/s10554-009-9499-1 19757151 PMC2795157

[32] Cannan C, Eitel I, Hare J, Kumar A, Friedrich M (2010) Hemorrhage in the myocardium following infarction. JACC Cardiovasc Imaging 3: 665–668. 10.1016/j.jcmg.2009.12.019 20541722

[33] Croisille P, Kim HW, Kim RJ (2012) Controversies in Cardiovascular MR Imaging: T2-weighted Imaging Should Not Be Used to Delineate the Area at Risk in Ischemic Myocardial Injury. Radiology 265: 12–22. 22993217 10.1148/radiol.12111769

[34] Eitel I, Kubusch K, Strohm O, Desch S, Mikami Y, de Waha S, et al (2011) Prognostic value and determinants of a hypointense infarct core in T2-weighted cardiac magnetic resonance in acute reperfused ST-elevation-myocardial infarction. Circ Cardiovasc Imaging 4: 354–362. 10.1161/CIRCIMAGING.110.960500 21518773

[35] Francone M, Carbone I, Agati L, Bucciarelli Ducci C, Mangia M, Iacucci I, et al (2011) Utility of T2-weighted short-tau inversion recovery (STIR) sequences in cardiac MRI: an overview of clinical applications in ischaemic and non-ischaemic heart disease. Radiol Med 116: 32–46. 10.1007/s11547-010-0594-0 20927650

[36] Ganame J, Messalli G, Dymarkowski S, Rademakers FE, Desmet W, Van de Werf F, et al (2009) Impact of myocardial haemorrhage on left ventricular function and remodelling in patients with reperfused acute myocardial infarction. Eur Heart J 30: 1440–1449. 10.1093/eurheartj/ehp093 19346229

[37] Lotan CS, Miller SK, Bouchard A, Cranney GB, Reeves RC, Bishop SP, et al (1990) Detection of intramyocardial hemorrhage using high-field proton (1H) nuclear magnetic resonance imaging. Cathet Cardiovasc Diagn 20: 205–211. 2163757 10.1002/ccd.1810200313

[38] Mather AN, Fairbairn TA, Ball SG, Greenwood JP, Plein S (2011) Reperfusion haemorrhage as determined by cardiovascular MRI is a predictor of adverse left ventricular remodelling and markers of late arrhythmic risk. Heart 97: 453–459. 10.1136/hrt.2010.202028 21051455

[39] Mikami Y, Sakuma H, Nagata M, Ishida M, Kurita T, Komuro I, et al (2009) Relation between signal intensity on T2-weighted MR images and presence of microvascular obstruction in patients with acute myocardial infarction. AJR Am J Roentgenol 193: W321–326. 10.2214/AJR.09.2335 19770302

[40] Porto I, Hamilton-Craig C, De Maria GL, Vergallo R, Cautilli G, Galiuto L, et al (2011) Quantitative Blush Evaluator accurately quantifies microvascular dysfunction in patients with ST-elevation myocardial infarction: comparison with cardiovascular magnetic resonance. Am Heart J 162: 372–381 e372. 10.1016/j.ahj.2011.04.014 21835300

[41] Kumar A, Green JD, Sykes JM, Ephrat P, Carson JJ, Mitchell AJ, et al (2011) Detection and quantification of myocardial reperfusion hemorrhage using T2*-weighted CMR. JACC Cardiovascular imaging 4: 1274–1283. 10.1016/j.jcmg.2011.08.016 22172784

[42] Ochiai K, Shimada T, Murakami Y, Ishibashi Y, Sano K, Kitamura J, et al (1999) Hemorrhagic myocardial infarction after coronary reperfusion detected in vivo by magnetic resonance imaging in humans: prevalence and clinical implications. J Cardiovasc Magn Reson 1: 247–256. 11550358 10.3109/10976649909088337

[43] Goldfarb JW, Hasan U, Zhao W, Han J (2014) Magnetic resonance susceptibility weighted phase imaging for the assessment of reperfusion intramyocardial hemorrhage. Magn Reson Med 71: 1210–1220. 23776036 10.1002/mrm.24747

[44] Haacke EM, Xu Y, Cheng YC, Reichenbach JR (2004) Susceptibility weighted imaging (SWI). Magn Reson Med 52: 612–618. 15334582 10.1002/mrm.20198

[45] Tong KA, Ashwal S, Obenaus A, Nickerson JP, Kido D, Haacke EM (2008) Susceptibility-weighted MR imaging: a review of clinical applications in children. AJNR Am J Neuroradiol 29: 9–17. 17925363 10.3174/ajnr.A0786 PMC8119104

[46] Quinn MP, Gati JS, Klassen LM, Lin AW, Bird JR, Leung SE, et al. (2013) Comparison of Multiecho Postprocessing Schemes for SWI with Use of Linear and Nonlinear Mask Functions. AJNR Am J Neuroradiol. 10.3174/ajnr.A3584 PMC7966483 23744694

[47] Gilbert G, Savard G, Bard C, Beaudoin G (2012) Quantitative comparison between a multiecho sequence and a single-echo sequence for susceptibility-weighted phase imaging. Magn Reson Imaging 30: 722–730. 10.1016/j.mri.2012.02.008 22459441

[48] Denk C, Rauscher A (2010) Susceptibility weighted imaging with multiple echoes. J Magn Reson Imaging 31: 185–191. 10.1002/jmri.21995 20027586

[49] Brainovich V, Sabatini U, Hagberg GE (2009) Advantages of using multiple-echo image combination and asymmetric triangular phase masking in magnetic resonance venography at 3 T. Magn Reson Imaging 27: 23–37. 10.1016/j.mri.2008.05.006 18599241

[50] Huber AM, Schoenberg SO, Hayes C, Spannagl B, Engelmann MG, Franz WM, et al (2005) Phase-sensitive inversion-recovery MR imaging in the detection of myocardial infarction. Radiology 237: 854–860. 16304107 10.1148/radiol.2373041483

[51] Roemer PB, Edelstein WA, Hayes CE, Souza SP, Mueller OM (1990) The NMR phased array. Magn Reson Med 16: 192–225. 2266841 10.1002/mrm.1910160203

[52] Cerqueira MD, Weissman NJ, Dilsizian V, Jacobs AK, Kaul S, Laskey WK, et al (2002) Standardized myocardial segmentation and nomenclature for tomographic imaging of the heart: a statement for healthcare professionals from the Cardiac Imaging Committee of the Council on Clinical Cardiology of the American Heart Association. Circulation 105: 539–542. 11815441 10.1161/hc0402.102975

[53] Wolff SD, Balaban RS (1997) Assessing contrast on MR images. Radiology 202: 25–29. 8988186 10.1148/radiology.202.1.8988186

[54] Amado LC, Gerber BL, Gupta SN, Rettmann DW, Szarf G, Schock R, et al (2004) Accurate and objective infarct sizing by contrast-enhanced magnetic resonance imaging in a canine myocardial infarction model. J Am Coll Cardiol 44: 2383–2389. 15607402 10.1016/j.jacc.2004.09.020

[55] Goldfarb JW, Arnold S, Roth M, Han J (2007) T1-weighted magnetic resonance imaging shows fatty deposition after myocardial infarction. Magn Reson Med 57: 828–834. 17457862 10.1002/mrm.21207

