# Correction: Imaging of Reperfused Intramyocardial Hemorrhage with Cardiovascular Magnetic Resonance Susceptibility Weighted Imaging (SWI)

**DOI:** 10.1371/journal.pone.0133112

**Published:** 2015-07-14

**Authors:** The PLOS ONE Staff

## Notice of Republication

This article was republished on June 22, 2015, to correct Figs 1–5 and 8–11, which were darker than intended. The publisher apologizes for the error. Please download this article again to view the corrected version.
